# Molecular Mechanism Underlying the Regulatory Effect of Vine Tea on Metabolic Syndrome by Targeting Redox Balance and Gut Microbiota

**DOI:** 10.3389/fnut.2022.802015

**Published:** 2022-02-17

**Authors:** Xixin Zhou, Ying Song, Chaoxi Zeng, Haowei Zhang, Chenghao Lv, Meng Shi, Si Qin

**Affiliations:** ^1^Department of Biological Sciences, College of Bioscience and Biotechnology, Hunan Agricultural University, Changsha, China; ^2^Lab of Food Function and Nutrigenomics, College of Food Science and Technology, Hunan Agricultural University, Changsha, China

**Keywords:** dihydromyricetin, metabolic syndrome, redox signaling pathway, gut microbiota, biotransformation

## Abstract

Metabolic syndrome (MS) is a metabolic disorder that arises from the increasing prevalence of obesity. The pathophysiology seems to be largely attributable to the imbalance of lipid and glucose metabolism, redox signaling pathways, and gut microbiota. The increased syndromes, such as type 2 diabetes and cardiovascular disease demands natural therapeutic attention for those at high risk. Vine tea, as a traditional medicinal and edible resource rich in flavonoids, especially for dihydromyricetin (DHM), exhibits promising health benefits on the intervention of MS, but the specific molecular mechanism has not been systematically elucidated. The present article aims to summarize the regulatory effects and biological targets of vine tea or DHM on MS, and analyze the underlying potential molecular mechanisms in cells, animals, and humans, mainly by regulating the redox associated signaling pathways, such as Nrf2, NF-κB, PI3K/IRS2/AKT, AMPK-PGC1α-SIRT1, SIRT3 pathways, and the crosstalk among them, and by targeting several key biomarkers. Moreover, vine tea extract or DHM has a positive impact on the modulation of intestinal microecology by upregulating the ratio of *Firmicutes*/*Bacteroidetes* (F/B) and increasing the relative abundance of *Akkermansia muciniphila*. Therefore, this review updated the latest important theoretical basis and molecular evidence for the development and application of vine tea in dietary functional products or drugs against MS and also imputed the future perspectives to clarify the deep mechanism among vine tea or DHM, redox associated signaling pathways, and gut microbiota.

## Introduction

Vine tea, also known as “Mei” tea, is the tender stem and leaves of *Ampelopsis grossedentata*, a plant of the Vitis family. Vine tea is commonly used as traditional medicine or functional tea by Tujia and Zhuang nationality in Hunan, Hubei, Guizhou, Jiangxi, and other provinces (1). Vine tea is containing abundant flavonoids, polysaccharides, alkaloids, other polyphenols, and active ingredients, thus presenting multiple biofunctions. The content of flavonoids in vine tea can reach about 45% (m/m), so it is called as “King of flavonoid-contained plant” (2). The flavonoids, separated and identified by Zhang et al. (3) from vine tea, are mainly dihydromyricetin (DHM), myricetin-3-O-beta-D-galactopyranoside, myricetin, and myricitrin, of which the most abundant is DHM, with the concentration of higher than 35% (m/m) (4).

In detail, this review aims to summarize the current state of vine tea research in metabolic syndrome (MS) and the recent studies in humans. The literature search took place in the PubMed, Web of Science, and CNKI databases by using the following queries: vine tea and metabolic syndrome, vine tea and diabetes, vine tea and obesity, vine tea and intestinal microorganism, *Ampelopsis grossedentata* and metabolic syndrome, *Ampelopsis grossedentata* and diabetes, *Ampelopsis grossedentata* and obesity, *Ampelopsis grossedentata* and intestinal microorganism, Dihydromyricetin and metabolic syndrome, Dihydromyricetin and diabetes, Dihydromyricetin and obesity, Dihydromyricetin and intestinal microorganism. The results were screened based on their titles, abstracts, and full-text availability, according to our inclusion criteria: MS, diabetes and obesity cell, animal or clinical studies evaluating vine tea or DHM. All non-English-related titles were excluded from the present review. Filter limits, such as text availability, article type, and publication date were not applied.

### MS and Vine Tea

Metabolic syndrome refers to the pathological disorders of protein, fat, carbohydrate, and other substances in the human body, which can cause a complex metabolic disorder syndrome and result in cardiovascular diseases and diabetes (5). The clinical symptoms of MS are hyperlipidemia, hyperglycemia, and central-obesity, which may induce cardiac, cerebral infarction, and arteriosclerosis in severe cases (6). The pathophysiology is probably largely attributable to insulin resistance with the excessive flux of fatty acids implicated and the proinflammatory state (7). The development of MS may associate with the change of gut microbes (8). According to the WHO statistics, about 17.9 million people die of cardiovascular diseases worldwide every year, while about 422 million patients with diabetes worldwide and 1.6 million people die each year from diabetes and its complications. In 2019, there were more than 400 million patients with dyslipidemia and 116.4 million diabetics in China. However, the current treatment of MS is mainly through drug counseling, which cannot delay the process of arteriosclerosis and prevent the occurrence of cardio-cerebral infarction (9).

A large number of studies have shown that the Chinese traditional characteristic substances, such as hawthorn (10), *Platycodon grandiflorum* (11), chrysanthemum (12), and other common dual-purpose substances rich in flavonoids are of great significance in intervening metabolic disorders. In recent years, studies have found that flavonoids in vine tea not only have anti-inflammatory and bactericidal (13), anti-tumor (14), anti-oxidation (15), and other biofunctions, but also have significant effects in reducing blood lipid (16), blood glucose (17), protecting the cardiovascular system, reducing whole blood and plasma viscosity, expanding blood vessels, and increasing blood flow velocity (18). The modulation effects of flavonoids from vine tea on MS may indeed be influenced by gut microbiota. Downregulation of the ratio of *Firmicutes* and *Bacteroidetes* (F/B) in the human gastrointestinal tract was observed to alleviate metabolic disorders. Studies have shown that the F/B value is upregulated in obese patients (19). The level of glucagon-like peptide 1 (GLP-1) can be increased with the decrease of F/B values in insulin resistant mice (20). These results suggest that vine tea and its extracts can interfere with MS by regulating the abundance and composition of gut microbes.

Thus, this article documented the digestion, absorption, and metabolism of vine tea and its main functional component DHM in the human body, and their intervention effects on abnormal glucose and lipid metabolism and the potential molecular mechanism in animals and human studies were also summarized, aiming to provide a reliable theoretical basis for in-depth research and development of vine tea and DHM in functional food and clinical application of human body.

### DHM From Vine Tea and Its Extraction

Dihydromyricetin, with a molecular weight of 320.25, is a white needle crystal, easily soluble in hot water, hot ethanol and acetone, slightly soluble in ethanol and methanol, very slightly soluble in ethyl acetate, insoluble in chloroform and petroleum ether. As a typical flavonoid compound, DHM has two chiral centers, so it is possible to obtain four potential enantiomers (21), which are vulnerable to environmental pH value and have poor stability. DHM is more stable under neutral and weakly acidic conditions (22). In addition, DHM produces an irreversible oxidation reaction when the ambient temperature exceeds 100°C (23). In fact, metal ions (such as Fe^3+^, Al^3+^, and Cu^2+^) affect the chemical stability of DHM (24).

Dihydromyricetin, as the highest content of flavonoids in vine tea, shows obvious differences from regions. Chang proposed that in Guangxi province, DHM content in young leaves of vine tea in Guilin and Cenxi was 3~15 times higher than that in Nanning (25). Zheng et al. had extracted DHM from vine tea from Hubei, Fujian, Yunnan, Guangxi, and Hunan in different ways, and the results showed that the extraction amount of DHM in vine tea from Yunnan province was the highest, which was 1.5 times that of Hubei province (26). In addition, different extraction methods were important factors. Studies showed that the DHM content in vine tea is about 21.86% under the conditions of 90°C, the solid-liquid ratio of 1:20 with the extraction time of 120 min (27). Under the conditions of 45–48°C, the solid-liquid ratio of 25 ml/g with the extraction time of 27–28 min, the content of DHM in ethanol extract is about 31.56% (28). Thus, it is very important to show the origin of vine tea while it was used to develop functional products and the extraction methods matter.

### Digestion, Absorption, Metabolism, and Toxicity of DHM

When the administration of DHM, most of the prototype compounds and metabolites are excreted from the body through feces, while a small number of prototype compounds are absorbed through the small intestine, enter the blood circulation through the portal vein and reach to the liver, which further generates phase II metabolites through glucuronidation, sulfation, and methylation, absorbed by our body or excreted with urine. The oral bioavailability of DHM is quite low because of the occurrence of a large number of biotransformation reactions in the gastrointestinal tract and blood (29). The peak concentration (Cmax) of DHM was only reached 21.63 ± 3.62 ng/ml after 2.67 h treatment with an oral dose of 20 mg/kg. The half-life is 3.70 ± 0.99 h, and the resident time is 5.98 ± 0.58 h (30). In addition, DHM is easily eliminated and degraded by methylation, sulfide, and reduction reactions after entering the blood (31). In the rat model, the Cmax was 165.67 ± 16.35 ng/ml, the half-life was 2.05 ± 0.52 h, and the residence time was only 2.62 ± 0.36 h after intravenous injection of 2 mg/kg DHM. The bioavailability of DHM in the oral group and the intravenous group was only 4.02% (30). When the blood concentration is 100 ng/ml, the cell contact concentration is about 0.3 μM, which is much lower than the actual cell treatment concentration (0.01–300 μM) (Table 1). This also explained why oral DHM products were not effective. Meanwhile, DHM solution is easy to be oxidized and acylated, limiting its application in functional food and clinical medicine (38).

**Table 1:** The effect and molecular mechanism of DHM on MS in cell model.

**Effect**	**Model**	**Drugs**	**Dose and time**	**Results**	**References**
Protect vascular endothelial cells	Vascular endothelia-l cell	DHM	37.5, 75, 150, 300 μM	Protect the Vascular endothelial cell 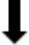 ; Blood clots 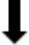 ; Bax/Bcl-2 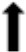	(32)
Improves insulin resistance	Insulin resistant cell	90% DHM	1*10^−2^-1*10^−5^ μM	GLUT4, Akt2 expression 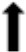 ; Adiponectin 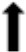	(33)
	Insulin resistance HepG2 cells	DHM	5, 10, 20, 40 μM	MDA 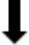 ; CAT, ATP, SOD 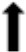	(34)
Enhance glucose uptake in adipocytes	Embryonic fibroblast 3T3-L1 cells	DHM	1, 3, 10 μM	p-PPARγ273, p-ERK 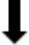 ; Adiponectin 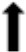 ; Insulin sensitivity 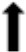	(35)
Improved inflammation induced by high glucose	PCl2	DHM	15 μM	PC12 apoptosis 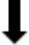 ; Bax, caspase-3, p-JNK 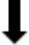 ; Bcl-2 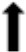	(36)
Relieves acute inflammation induced by LPS	RAW264.7	DHM	37.5, 75, 150, 300 μM	NO release 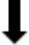 ; TNF-α, IL-6, IL-1β 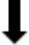 ; IL-10 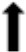	(37)

*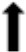, stands for up-regulation; 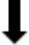, stands for down-regulation*.

Structure modification of DHM was regarded as an effective way to improve its stability and functionality. DHM esterification, when formed by the reaction with acetic anhydride, increased its solubility in peanut oil by 4~6 times as well as its antioxidant activity (39). DHM reacts with perchloric acid and acetic anhydride to form DHM acetylated derivatives. Pharmacokinetic data showed that the half-life of DHM acetylated derivatives was two times that of DHM monomer, and the residence time *in vivo* was longer. The apparent volume of distribution (Vd) showed that the acetylated derivatives had a wider distribution area *in vivo* and higher stability (40). In addition, the water solubility of DHM glycosides increased 89 times and the Browning resistance increased 14.5 times (41). DHM can increase its solubility in water from 0.74 to 53.64 mg/ml by forming an inclusion complex with hydroxypropyl-β-cyclodextrin, and the thermal decomposition reaction of DHM can also be delayed by forming inclusion (42).

Vine tea had no obvious toxic side effects on rats, mice, rabbits, and domestic dogs, which could be used as a non-toxic food raw material. The administration dose at 250 mg/kg to mice (43), at 400 mg/kg to broilers (44), at 150 mg/kg to domestic dogs (45), and intragastric administration of 93 mg/kg DHM to rabbits have no obvious toxicological side effects on body weight, food intake, blood physiological and biochemical indicators, organ weight, histopathological indicators, and skin allergy indicators (46). Thus, vine tea/DHM was regarded as safe with multiple functionalities.

### Application of Vine Tea and Its Functional Components in Medicine and Food

Vine tea and DHM related products have gathered the attention of the scientific and industrial community due to their various possible applications. The health food of vine tea approved by China's Food and Drug Administration, such as “Kubao Zhishukang Capsule (12.7 g total flavonoids/100 g)” and “Jinqi vine tea (3.5 g total flavonoids/100 g)” which have the effect on lowering blood lipid, and “Xihuang Bazhen tea” which was found to enhance the immune function and protect the function of chemical liver injury. According to the document of “announcement on three new food raw materials such as the leaves of vine tea” (No. 16, 2013) adopted by the national health and Family Planning Commission, vine tea is legal to use as raw material to develop common food (47). In addition, the current food production principles (SC) classification of vine tea support it to be used as common food or common food base material to develop into “tea and related products.” All these enable a steadily growing of bag brewed vine tea (48), compound vine-tuo tea (49), “Jinhua vine tea (50),” and vine tea related drinks (51). In addition, products, such as camellia noodles (52), camellia gummy candy, and camellia jelly have been put into the market (53, 54). Although several rattan tea products have been developed, research on their safety and function is still not clear.

## The Potential Molecular Mechanisms of Vine Tea and DHM Underlying the Interventional Effect on MS in Cell and Animal Models

Metabolic syndrome is a common metabolic disorder defined by a constellation of interconnected physiological, biochemical, clinical, and metabolic factors, which directly increases the risk of cardiovascular disease, type 2 diabetes mellitus (T2DM), and cause mortality, seriously threatening human health and quality of life (55). Due to modern patterns of life style, such as surplus energy intake and sedentary life habits, the incidence of MS is increasing and tends to be prevalent in young people. Vine tea and its main functional component DHM were used to treat obesity model rats (10–500 mg/kg/day for 6–24 weeks) and diabetes model rats (30–500 mg/kg/day for 2–16 weeks) and inflammatory model mice (57.5–500 mg/kg/day for 1–120 days), as shown in Table 2, mainly through the targeting redox signaling pathways, such as Nrf2 antioxidant pathway, NF-κB inflammatory pathway, PI3K/IRS2/AKT insulin resistance pathway, and AMPK-PGC1α-SIRT1, SIRT3 energy metabolism related pathway.

**Table 2:** The effect and molecular mechanism of dihydromyricetin (DHM) on metabolic syndrome (MS) in animals.

**Effect**	**Model**	**Drugs**	**Dose and time**	**Results**	**References**
Relieves atherosclerosis	Atherosclerotic mice lacking LDL receptors	DHM	Intragastric gavage with 250, 500 mg/kg/day 8 weeks	TNF-α, IL-6, MDA 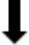 ; GSH-x, CAT 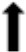	(16)
Reduce hematic fat	Hyperlipidemia rats	Vine tea flavonoids of alcohol extraction	Intragastric gavage with 100, 200, 400 mg/kg 30 days	Cholesterol synthesis 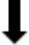 ; TC, TG, MDA 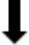 ; GSH, SOD, CAT 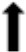 ; Lipid vacuole area in hepatocytes 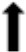 ; Phospholipids 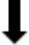	(56)
	ApoE(-/-)mice	98% DHM	Intragastric gavage with 50, 100 mg/kg/day 12 weeks	Hepatic steatosis 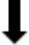 ; ALT, AST, MDA 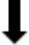 ; CAT, SOD 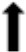 ; p-AMPK 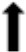	(57)
Reduce hematic fat	Fat mice	98% DHM	Intragastric gavage with 100, 300, 500 mg/kg/day 6 weeks	TC, TG, LDL-C, MDA 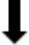 ; HDL-C, SOD 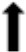	(58)
Reduce hematic fat	High-fat mice	98% DHM	Intragastric gavage with 125, 250 mg/kg/day 16 weeks	TG, TC, LDL-C, ALT, AST, MDA 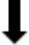 ; Lipid droplets 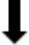 ; HDL-C, SOD 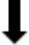 ; IL-6, IL-8, TNFα 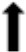 ; SIRT1 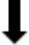 ; AMPKmRNA 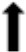 ; SREBP1 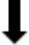 ; ACC1, FAS 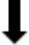 ; PPARαCPT1 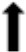	(59)
Promote the reverse transport of cholesterol	ApoE(-/-)mice	DHM	Intragastric gavage with 250, 500 mg/kg/day 8 weeks	Lipid deposition and cholesterol 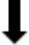 ; TG, TC in liver 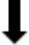 ; ABCG5, ABCG8, CYP7A1mRNA 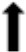 ; ABCG5, ABCG8 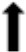	(60)
Relieves atherosclerosis	Atherosclerotic rats	97.65% DHM	Intragastric gavage with 10, 40 mg/kg 24 weeks	TG, TC, LDL 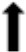 ; HDL-C, MDA 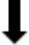 ; SOD 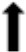	(61)
	High lipid induces atherosclerosis in rabbits	DHM	Intragastric gavage with 10, 40 mg/kg/day 24 weeks	HDL, LDL, TC 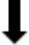 ; AS plaque 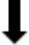 ; Becilin−1, LC3-II, LC3-II/LC3-I 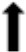 ; p62 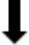	(62)
Reduce hematic fat	Fat mice	DHM	Intragastric gavage with 125, 250 mg/kg/day 16 weeks	UCP1 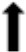 ; WAT brown 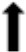	(63)
Promotes Browning of adipose tissue	Fat mice	DHM	Intragastric gavage with 125, 250 mg/kg/day 16 weeks	UCP1, Prdm16 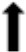 ; AMPK, PGC1α, Sirt1mRNA 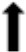	(64)
Improve alcoholic fatty liver disease	High-fat SIRT3 knockout mice	DHM	Intragastric gavage with 300 mg/kg/day 12 weeks	Hepatic steatosis, Inflammation, fat cavitation 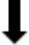 ; SOD2 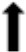	(65)
Reduce blood sugar	Diabetic mice	95.1% DHM	Intragastric gavage with 50, 100, 150 mg/kg/day 4 weeks	Blood sugar 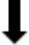 ; Insulin 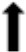 ; TC, TG, LDL 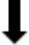 ; HDL 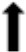	(66)
Increased insulin sensitivity	Diabetic mice	DHM	Intragastric gavage with 50, 100, 200 mg/kg/day 8 weeks	Insulin level 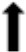 ; Serum adiponectin level 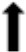 ; p-PPARγ level 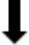	(67)
Alleviate diabetic cognitive dysfunction	Diabetic mice	DHM	Intragastric gavage with 125, 250 mg/kg/day 16 weeks	Oxidative stress in the hippocampus of T2DM mice 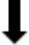	(67)
Improves insulin resistance	Type 2 diabetic mice	98% DHM, Vine tea extract of decocting concentrated	Intragastric gavage with DHM(100 mg/kg/day), Vine tea(50, 100, 200 mg/kg/day) 4 weeks	Blood sugar 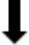 ; Serum insulin, HOMA-IR 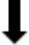 ; p-Akt, FGF21, p-AMPK in liver tissue 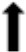 ; Serum insulin C peptide 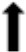	(68)
Increased insulin sensitivity	High fat induced SD rats	DHM	Intragastric gavage with 100, 200, 400 mg/kg/day 8 weeks	Insulin sensitivity 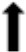 ; Liver fat accumulation 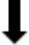	(69)
Increased insulin sensitivity	Type 2 diabetic mice	98% DHM	Intragastric gavage with 50 mg/kg/day 12 weeks	p-IRS-1, p-AKT 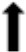 ; Insulin sensitivity 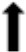 ; LC3-II 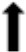 ; p62 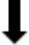	(70)
Alleviate diabetic cognitive dysfunction	Diabetic mice	DHM	Intragastric gavage with 125, 250, 500 mg/kg/day 16 weeks	PI3K, Akt, CREB, BDNF 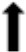 ; Cognitive function 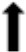	(71)
	Streptozotocin (STZ) -induced diabetic mice	DHM	Intragastric gavage with 125, 250 mg/kg/day 16 weeks	BDNF 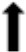 ; Blood sugar 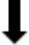 ; Cognitive function in type 2 diabetic mice 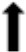	(71)
	Diabetic and DP mice	DHM	Intraperitoneal 30 mg/kg/day 14days	Hippocampal P2X7 receptor 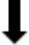	(72)
Reduce the inflammatory response of focal brain I/R injury	Mice with focal brain I/R injury	DHM	Intragastric gavage with 500 mg/kg/day 10 days	TNF-α 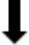 ; 5-LOX, LTB4, CysLTs 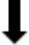	(73)
Relieves cognitive impairment and inflammation	Lead poisoned mouse	DHM	Intragastric gavage with 125, 250 mg/kg/day 12 weeks	CAT, SOD 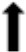 ; Caspase3, Bax 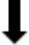 ; Bcl-2 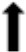 ; TNF-α, IL-1β, NF-κB p65 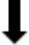 ; Inhibition of p38 signaling	(74)
Improve the inflammation	High fat obese mice	DHM	Intragastric gavage with 50, 100 mg/kg/day 16 weeks	mRNA expression of TNF-α, IL-1β, IL-6 and IL-10 in colon tissue 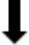	(75)
Reduces oxidative stress and inflammation	Diabetic rat	DHM	Intragastric gavage with 160, 320, 480 mg/kg/day 16 weeks	Blood sugar, BUN, SCr 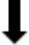 ; SOD, HO-1, GSH-Px 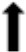 ; MDA, Nrf2 trans-nuclear 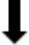 ; TNF-α, IL-6, IL-1β 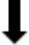 ; NF-kB 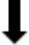 ; Expression of IKBα protein in kidney 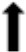	(76)
Alleviate diabetic nephropathy and renal fibrosis	Streptozotocin (STZ) -induced diabetic mice	DHM	Intragastric gavage with 125, 250, 500 mg/kg/day 12 weeks	24 h-Pro, (BUN), Scr 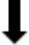 ; TGF-β1, Smad2, Smad 7 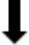	(77)
Relieves acute inflammation induced by LPS	Acute inflammatory mice	DHM	Intraperitoneal 57.5, 115, 230, 460 mg/kg	TNF-α, IL-6, IL-1β 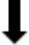 ; IL-10 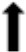	(37)
Relieved TPA induced ear swelling	Ear swelling mouse	DHM	External application 230, 460 mg/kg	Inflammatory symptoms 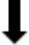	(37)

*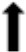, stands for up-regulation; 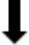, stands for down-regulation*.

### Regulation on Blood Lipid

Vine tea and DHM play an important role in reducing blood lipid and blood glucose. DHM can significantly inhibit the biosynthesis of intracellular cholesterol in the high-fat model, decrease the levels of triglyceride (TG), total cholesterol (TC), and low-density lipoprotein (LDL-C), and increase the levels of high-density lipoprotein (HDL-C), superoxide dismutase (SOD), and catalase (CAT). Moreover, vine tea and DHM can lower the expression of alanine transaminase (ALT) and aspartate aminotransferase (AST) and reduce the level of malondialdehyde (MDA) (56, 57). In hepatocytes of obese mice treated with DHM, the lipid droplet status was significantly improved, the fat vacuole area was reduced, the expression of liver lipid anabolism-related genes (SREBP1, ACC1, and FAS) was decreased, and the expressions of liver lipid metabolism related genes (PPARα and CPT1) were upregulated (58). In high-fat ApoE (-/-) mice, DHM showed an effect on lowering the hepatic steatosis, regulating the expression of antioxidant enzymes, inhibiting the expression of adenylate phosphorylated protein kinase (p-AMPK) in liver tissue to inhibit hepatocyte apoptosis, protect liver tissue, alleviate liver injury, and improve the metabolic abnormalities in high-fat mice (59). In addition, DHM significantly reduced the lipid deposition and cholesterol content in mouse peritoneal macrophages, increase the expressions of ABCG5, ABCG8, and CYP7A1mRNA, promote the decomposition and excrement of cholesterol in the liver, and increases the protein expressions of ABCG5 and ABCG8 in the small intestine in mice (60). In atherosclerosis (AS) model, the mRNA expression of platelet phospholipase A2 of group IIA (sPLA2-IIA) was significantly decreased, the expressions of Beclin-1 and LC3-II were significantly increased, and the expression of p62 was significantly downregulated; while diffuse uplift in thoracic aorta wall, the proportion of AS plaque area and the degree of common carotid artery lesions were all reduced (61, 62). In high-fat obese mice, DHM reduced the size of shoulder adipose tissue cells and increased the expressions of UCP1 and Prdm16 in white adipose tissue (WAT) (63). The mRNA and protein expressions of adenylate activated protein kinase (AMPK), peroxisome proliferation-activated receptor γ (PGC1α), and silent message regulator (Sirt1) were increased in subscapular adipose tissue of mice. It is speculated that DHM can promote adipocyte brown-resistance to obesity through the AMPK-PGC1α-SIRT1 pathway (64). In addition, DHM-induced activation of SIRT3 can promote the expression of mitochondrial respiratory chain enzyme complex in hepatocytes, increase SOD-2-mediated mitochondrial antioxidant capacity, and inhibit oxidative stress in hepatocytes. These results suggested that DHM may inhibit the oxidative stress in hepatocytes by upregulating SIRT3 expression by activating the AMPK/PGC1α pathway and promoting mitochondrial translocation (65).

### Regulation on Blood Glucose and Insulin Resistance

In diabetic models, DHM decrease the contents of TC, TG, and LDL-C while HDL-C and serum adiponectin were enhanced (66). Insulin resistance is commonly occurring in patients with diabetes and other metabolic disorders. Proinflammatory factor tumor necrosis factor-α (TNF-α) can inhibit serine phosphorylation of insulin receptor substrate-1 (IRS-1) by reducing the level of guanylate kinase related protein 42, thereby inhibiting the downstream glycogen synthesis and glucose transporter 4 (GLUT-4) activity to cause insulin resistance (78). At the same time, it can also induce hepatic steatosis by activating sterol regulatory element binding protein-1c (SREBP-1c), an important transcription factor of lipid synthesis gene expression, resulting in increased fatty acid synthesis and excessive accumulation of triglycerides (79). The treatment with DHM showed more intact islet tissue and increased β cells and insulin level, with the decrease of blood glucose. The expression levels of p-Akt, p-IRS-1, and p-AMPK in liver tissue were significantly increased, and insulin sensitivity was improved (67–69). In the cell model, it can also reduce the blood glucose level, upregulate the ratio of Bax/Bcl-2, the expression of GLUT4 and Akt2, and downregulate the phosphorylation of peroxisome proliferator- activated receptor γ (PPAR γ) and ERK to improve the insulin sensitivity (32–35).

Autophagy is a type of programmed apoptosis, which can regulate the renewal of new and old cells, and plays an important role in maintaining the intracellular homeostasis in response to intracellular stress (80). The expression of autophagy related genes (ATG14, RB1CC1/FIP200, GABARAPL1, and WIPI1) and protein LC3BII and ATG5 in skeletal muscle in patients with T2DM following in severe insulin resistance are downregulated (81). Impaired autophagy will further aggravate the metabolic disorder associated with diabetes in insulin target tissues (such as liver, skeletal muscle, and adipose tissue) and pancreatic β cells (82). In mouse skeletal muscle tissue, DHM can upregulate the expression of autophagy factor LC3-II and downregulate the expression of p62, and the number of autophagosomes after induction depends on SIRT3 regulation, suggesting that DHM may regulate SIRT3 induced autophagy in skeletal muscle through AMPK-PGC1α pathway to improve the insulin sensitivity (70).

Dihydromyricetin upregulated the expression of autophagy factor LC3-II and downregulated the expression of p62 in skeletal muscle, and the number of autophagosomes after induction was dependent on SIRT3 regulation, suggesting that DHM may improve the insulin sensitivity by regulating SIRT3-induced autophagy in skeletal muscle through AMPK-PGC1α pathway (70). It is well known that patients with diabetes are associated with many complications, among which neurodegenerative diseases, such as Alzheimer's disease (AD) have a great impact on the lives of patients and their families. In mice with T2DM, DHM inhibits oxidative stress in the hippocampus (66), increases the protein expression of phosphatidylinositol 3-kinase (PI3K), Akt, and brain-derived neurotrophic factor (BDNF) in the hippocampus, and stimulate improvement of cognitive dysfunction in T2DM mice (71). The expression of the P2X7 receptor, a family of ATP-gated ions, was downregulated in the hippocampus of mice, and diabetic neuropathic pain (DNP) and depression (DP) symptoms were both significantly improved (72).

### Regulation on Redox and Inflammatory Associated Signaling Pathways

The development of obesity and type 2 diabetes is closely related to mitochondrial dysfunction and oxidative stress. Ectopic lipid deposition results from insulin resistance, causing mitochondrial function reduction and oxidative imbalance. Oxidative stress is able to destroy the liver antioxidant enzyme system, resulting in hepatocyte injury and abnormal liver function (83, 84). From the point of antioxidant enzyme, DHM can upregulate the levels of SOD and CAT (56, 57). In addition, Liang found that DHM could alleviate vascular wall thickening, endothelial roughness, and media smooth muscle fiber hyperplasia in rats (61). DHM significantly reduces serum C-reactive protein (CRP), serum TNF-α, and interleukin (IL-1) levels in mice with focal brain I/R injury (73). DHM can downregulate the expressions of apoptotic protease 3 (Caspase3), Bcl-2 family protein (Bax), and upregulate the expression of Bcl-2 protein in an inflammatory mice model. DHM decreased the level of TNF-α and interleukin-1 β (IL-1β), followed by the decreased translocation of nuclear transcription factor NF-κB and inhibition of MAPK family protein p38. It is suggested that DHM may alleviate oxidative stress and apoptosis through the MAPK pathway (36, 74, 75). DHM reduced the nuclear factor erythroid-2-related factor 2 (Nrf2) nuclear translocation, upregulated downstream heme oxygenase (HO-1) expression in diabetic rats (76). Downregulation of NF-κB expression, pro-inflammatory factor interleukin-6 (IL-6), and interleukin-8 (IL-8) levels, and upregulation of anti-inflammatory factor interleukin-10 (IL-10) by DHM treatment were also observed (37, 77).

### Regulation on Intestinal Microbiota

Gut microbiota plays a crucial role in human health. The alteration of the composition of gut microbiotas is related to nutrient acquisition and energy regulation. Studies have shown that human intestinal flora can produce DHM metabolites *in vitro* and confirmed that DHM can be reduced and dehydroxylated to form metabolites under the action of intestinal microbes (24). Furthermore, DHM can regulate the composition of intestinal microflora by changing the richness and diversity of intestinal microflora (85). The ratio of F/B was significantly reduced by DHM in the gut microbes, and its decrease is positively correlated with the reduction of the obesity risk (86). Xie et al. confirmed that vine tea extract could reduce the proportion of F/B and increase the relative abundance of *Akkermansia ruminoccoccus* in high-fat diet mice (87). It inhibits the reproduction of *Enterobacteriaceae*, interferes with the relative abundance of intestinal microorganisms, prevents the imbalance of gut microbes, and improves non-alcoholic fatty liver disease (NAFLD). Multiple studies have shown that the relative abundance of *Bacteroides, Ruminoccoccus*, and *Akkermansia* were significantly changed in patients with NAFLD, and the serum endotoxin was increased, which induced the occurrence of insulin resistance and intestinal inflammation (88). Moreover, the decreased expression of ZO-1 in the inner wall of the small intestine leads to changes in intestinal permeability and a large number of enterogenic bacteria and metabolites enter the blood and the liver tissue, increasing the burden of the liver and leading to hepatotoxicity (89). DHM restores the ratio imbalance between *Firmicutes* and *Bacteroidetes* induced by HFD and upregulates the relative abundance of *Akkermansia* and *Prevotella*. Additionally, DHM significantly reduces the serum endotoxin level and increases the level of ZO-1 protein in intestinal tissue (87).

Despite the evidence indicating the interaction between DHM and gut microbes, the current studies focus on the effect of DHM on gut flora and the biotransformation of DHM under gut microbiota. The specific gut microbes responsible for the metabolism of DHM are still unknown. At present, 8 kinds of DHM metabolites in the urine and fecal of rats have been isolated and identified, but no follow-up investigations have been conducted (29), and the effects of DHM on the intestinal immunity, intestinal function, and inflammatory development after changing the composition of gut microbes have not been fully clarified (90).

## Interventional Effects of Vine Tea and DHM on Human MS

According to our literature search strategy, there are only two clinical studies published on vine tea or DHM against human MS up to date (Table 3). Ran divided the 80 patients with T2DM into vine tea group (970 mg/day, DHM) group and placebo group for a month in a double-blind clinical trial to monitor the blood glucose, insulin, blood lipids, and renal function indexes (91). Compared with the placebo, the levels of fasting blood glucose, glycated albumin, bladder C, and RBP-4 were significantly decreased in the vine tea group (91). The limitations of this first clinical research are the small popularity especially on elder people and the lack of experiment design on people who only received insulin or metformin. TNF-α, fibroblast growth factor 21 (FGF21), and insulin homeostasis assessment index were significantly reduced in patients treated with DHM (300 mg/day) in NAFLD (92). However, the parameters were only determined at the end of the intervention as it is of great importance to know the changes over time. The clinical research of DHM was very limited as one challenge is the low bioavailability of DHM. The other reason is the “dose-time-toxicity-functionality” of DHM, which is still unknown.

**Table 3:** Effects of DHM on MS in the human body.

**Effect**	**Model**	**Drugs**	**Dose and time**	**Results**	**References**
Fall blood sugar	Patients with type 2 diabetes	DHM	Oral 970 mg/day 4 weeks	GLU, GA, Cystatin C, RBP4 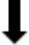	(91)
Anti-inflammatory	Patients with non-alcoholic fatty liver disease	DHM	Oral 300 mg/day 12 weeks	ALT, AST, Y-GT, LDL-c, ApoB, TNF-a, FGF21 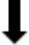	(92)

*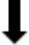, stands for down-regulation*.

## Discussion and Conclusions

**Figure 1:**
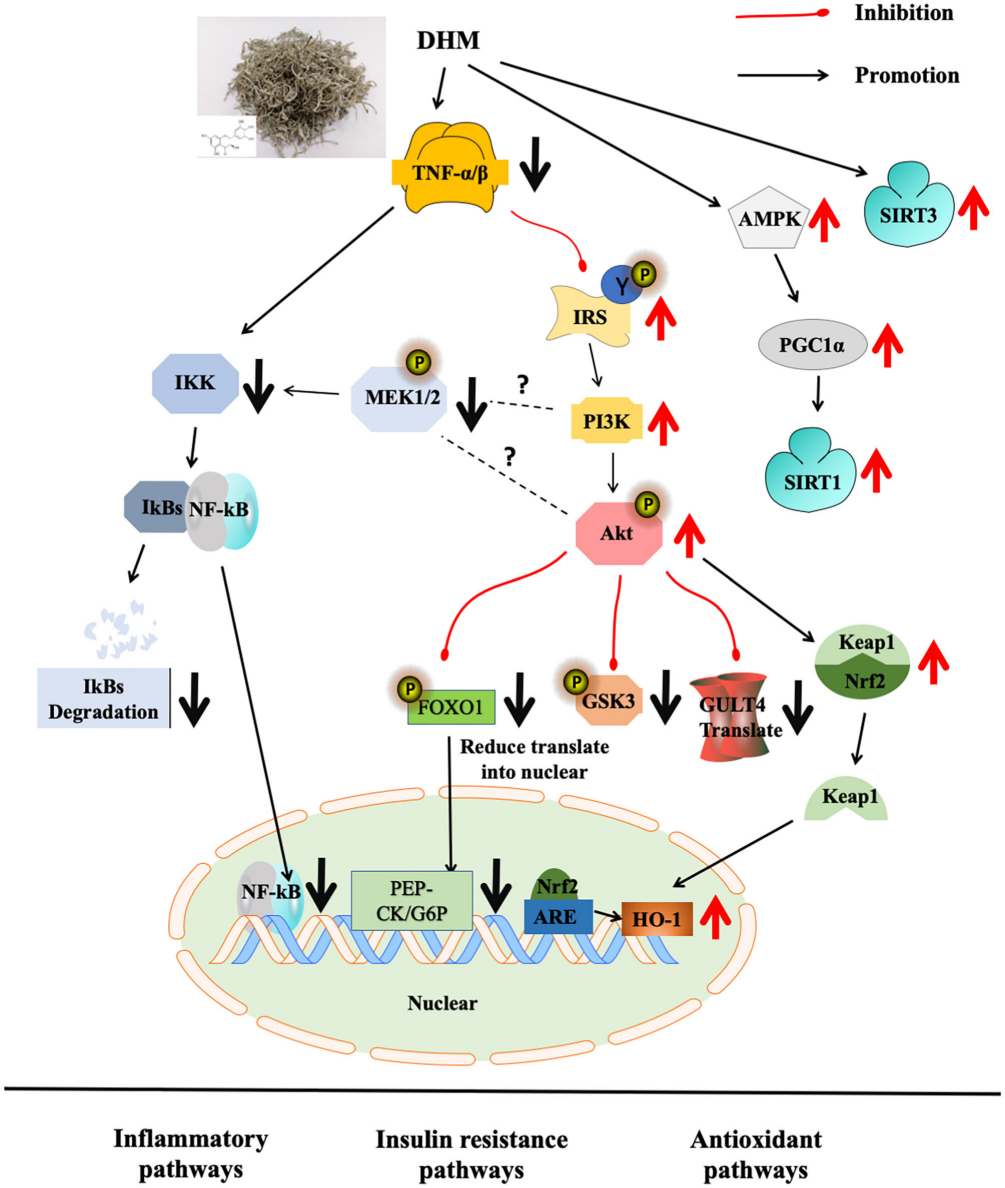
Molecular mechanism of DHM on abnormal glucose and lipid metabolism.

Dihydromyricetin, as the main functional compound of vine tea, is a plant resource with homologous medicinal and food characteristics in China with multiple bio-functions. Vine tea and DHM showed effects on regulating MS and their molecular pathways, such as Nrf2/ARE, NF-κB/JNK, AMPK/PGC1α/Sirt1 in obesity and diabetes models in animal models (Figure 1). It would be interesting to discuss the intervention mechanism of MS by combining the oxidative stress and inflammatory pathway with insulin resistance and adipose cell browning in the future.

However, vine tea species should be clarified when used as raw material for functional food development due to the huge variation of DHM content among the species. Additionally, the poor water solubility of DHM leads to its low membrane permeability and bioavailability, which limited the wide application of vine tea. Therefore, improving the bioavailability of DHM by using molecular modification technology and encapsulation technology is the crucial step for the deep development of vine tea and its related products. Interestingly, the existing data of animal models showed that the cell contact concentration of DHM was much lower than the treatment concentration in the cell model, which might be one of the reasons for the poor results obtained from animal experiments. The effect of DHM with high purity of 98% on animal MS was not as good as that of vine tea extract with 60% of DHM, which may be related to the complex form of DHM in less pure extracts. Micro-nano systems, such as polysaccharides, modified starch, and Chinese medicine decocting may protect the biological activity and structural stability of flavonoids. Thus, forming a complex or using an encapsulation technique would be a promising way to improve the bioavailability of DHM.

**Figure 2:**
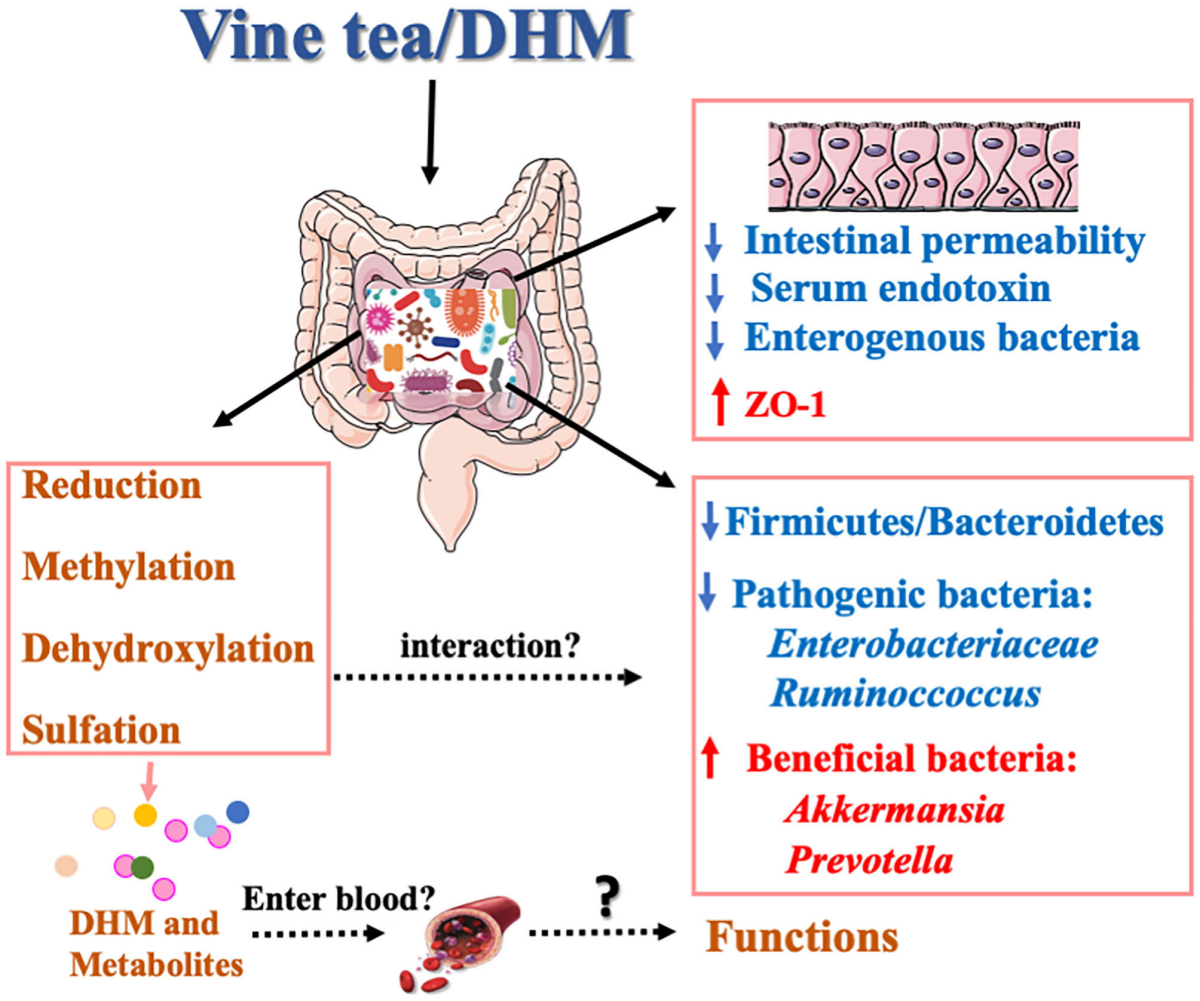
Potential mechanism of interaction between DHM and gut microbiota.

In addition, considering the different doses ranging from 10 to 500 mg/kg/day were applied in different animal models, it could be very interesting in the future to narrow down these differences and establish a safe range of DHM intake for humans. Sufficient data regarding of “dose-time-toxicity-function” of DHM from the long-term investigation will bring light to its application. In regards to the published articles, there were a lot of studies that had no purity results, which should be mentioned for future study. Although the interaction mechanism of gut microbiota and DHM has growing attraction in recent times (Figure 2), there is still a lot of work remaining to reveal the effects of DHM on the species structure and richness of gut microbiota and the intervention mechanism of its metabolites on MS, especially on whether these metabolites were absorbed in the blood and enter to the body circulation.

In the future, we can focus on DHM's highly enriched vine tea species, and improve the bioavailability of DHM. The deeper molecular mechanism, analyzed by multiomics to find the potential targeted protein and gene, as well as the biomarker of DHM on blood and gut were worth studying. Furthermore, more human clinical trials should be conducted to verify its function. Considering the availability of vine tea resources and its excellent health promoting effects, it would be very beneficial to have more vine tea related products entering foods, medicines, and clinical use.

## Author Contributions

XZ, YS, CL, and HZ wrote the first draft of the manuscript. YS, SQ, CZ, CL, and MS wrote sections of the manuscript. All authors contributed to manuscript revision, read, and approved the submitted version.

## Funding

This study was partially supported by the National Key Research and Development Program of China (2019YFC1604903), the Natural Science Foundation of China (31101268), and the Natural Science Foundation of Hunan Province (2019JJ40121 and 2019JJ40132).

## Conflict of Interest

The authors declare that the research was conducted in the absence of any commercial or financial relationships that could be construed as a potential conflict of interest.

## Publisher's Note

All claims expressed in this article are solely those of the authors and do not necessarily represent those of their affiliated organizations, or those of the publisher, the editors and the reviewers. Any product that may be evaluated in this article, or claim that may be made by its manufacturer, is not guaranteed or endorsed by the publisher.
